# Correlation of sonographic with intraoperative findings in laparoscopic managed ectopic pregnancies, a 10-year synopsis: a restrospective observational study

**DOI:** 10.1186/s12884-024-06441-y

**Published:** 2024-04-20

**Authors:** Steve Kyende Mutiso

**Affiliations:** Department of Obstetrics and Gynaecology, Aga-Khan University, P.O. Box 30270-00100, Nairobi, Kenya

**Keywords:** Ectopic pregnancy, Surgery, Ultrasound, Location, Correlation

## Abstract

**Background:**

Ectopic pregnancies (EP) are a common pregnancy complication that’s associated with significant morbidity and rarely mortality if not managed properly. Ultrasound examination forms the cornerstone of diagnosis of EP with some sonographic features occasionally not correlating with intraoperative findings. We set out to conduct an audit of EP managed surgically at our hospital for a 10-year period and discern the correlation and prediction of sonographic findings to intraoperative findings.

**Methods:**

This study was designed as a Retrospective Observational Study based at the Aga Khan University Hospital (AKUH). Study population was all women admitted to AKUH with a diagnosis of ectopic pregnancy that was surgically managed between the period of January 1st 2011 to December 31st 2020. Analysis of data was done against a pre-set checklist. Descriptive statistics for continuous variables was calculated and tabulated in graphs and tables. SPSS version 22 was used for analysis of data.

**Results:**

A total of 337 patients in this study had ultrasound findings. 99.7% (*n* = 336) of these patients had an intraoperatively confirmed EP. The commonest ultrasound finding was an adnexal mass in 97.1% (*n* = 309) of patients. These were confirmed surgically in 290 patients at the following locations: 76.6% (*n* = 222) were ampullary in location; 10.7% (*n* = 31) were fimbrial in location; 8.6%(*n* = 25) were isthmic in location; 2.4%(*n* = 7) were interstitial in location; 1%(*n* = 3) were abdominal in location; while 0.3% were located in the ovary(*n* = 1) or round ligament(*n* = 1) each. Interstitial EP on ultrasound were all (100%) confirmed in the same location intraoperatively, with ampullary EP also correlating fairly well with intraoperative location (75%). The distribution of location in the minor hemoperitoneum (HP) versus major HP groups were similar except for interstitial EP that increased from 1.4% in the minor HP group to 9.5% in the major HP group.

**Conclusion:**

In conclusion, ultrasonography still represents the best imaging modality for EP. The most common finding is usually an adnexal mass with no specific location. Most (99.7%) of the patients with this sonographic finding usually have a confirmed EP. Interstitial EP are the most well localized with ultrasound followed by ampullary EP. Furthermore, the presence of major (> 500mls) hemoperitoneum may act as an adjunct for diagnosis of an interstitial EP.

## Background

Ectopic Pregnancies(EP) are a common and significant cause of pregnancy related morbidity [1]. Ultrasound examination forms the cornerstone of diagnosis of EP with combination with clinical features and laboratory findings [2]. In this combination, the specificity of diagnosis EP is above 95% [3].

However, sonographic features may not correlate with intraoperative findings in about 10% of patients with some diagnostic laparoscopies for EP being negative [4]. Moreover, other sonographic findings such as the location or size of the EP may not correlate to intraoperative findings leading to a change of surgical strategy intraoperatively [5]. They also may not correlate well to other findings such as the volume of hemoperitoneum and even presence of an EP [4, 6].

We set out to conduct an audit of EP managed surgically at our hospital for a 10-year period and discern the correlation and prediction of sonographic findings to intraoperative findings.

## Methods

### Study setting and design

This study was designed as a Retrospective Observational study based at the Aga Khan University Hospital (AKUH). Study population was all women admitted to AKUH with a diagnosis of ectopic pregnancy that was surgically managed between the period of January 1st 2011 to December 31st 2020. Data was collected from clinical records during admission at AKUH. All patients were subjected to a full history taking and physical examination and laboratory tests including complete blood count, qualitative or quantitative Beta-human chorionic gonadotrophin (HCG), transvaginal and/or transabdominal ultrasound with determination of adnexal mass if present and presence and quantification of intraperitoneal haemorrhage. This was as per the management protocols at AKUH.

### Study procedures

#### Selection criteria

Inclusion Criteria will be all women diagnosed with ectopic pregnancies that were surgically managed at the AKUH that fulfilled the criteria for surgical management in accordance to the AKUH Early Pregnancy Bleeding protocol.

Exclusion criteria will be:

- Patients not treated at the Aga Khan University Hospital.
- Patients treated with any other methods i.e. conservative or medical.
- Patients with a heterotopic pregnancy.
- Patients files with missing data.

### Study tools

A data collection tool was designed incorporating pre-operative ultrasound findings, intraoperative findings, postoperative complications and costs attributed to the surgery. Standards of best practice is to be in accordance to the Royal College of Obstetricians and Gynaecologists Green Top Guideline, Number 21 [7].

### Data analysis and management

Analysis of data was done against a pre-set checklist. Descriptive statistics for continuous variables was calculated and tabulated in graphs and tables. These included: location of EP on ultrasound; intraoperative findings & volume of hemoperitoneum. Linear regression was done to ascertain whether the size of mass identified correlated with the volume of hemoperitoneum with the p value set at 0.005.

Statistical Package for Social Sciences (SPSS) version 22 was used for analysis of data.

The data collected was de-identified to maintain privacy and confidentiality. Collected data did not include patients name to safeguard patients’ privacy. The files were accessed at medical records and did not leave the custody of the medical records office. Data was collected using a data collection tool designed from the database file. Data was then entered directly into the database document created for use in this audit. The primary investigator (PI) was the sole custodian of this document and which was submitted to the research office at the end of the audit for archiving as with other studies. The hard copies of the data collection tools were also submitted to the research office for archiving. A back up copy of the document was kept on an online data cloud that will be password protected and accessible to the PI.

## Results

**Fig. 1:**
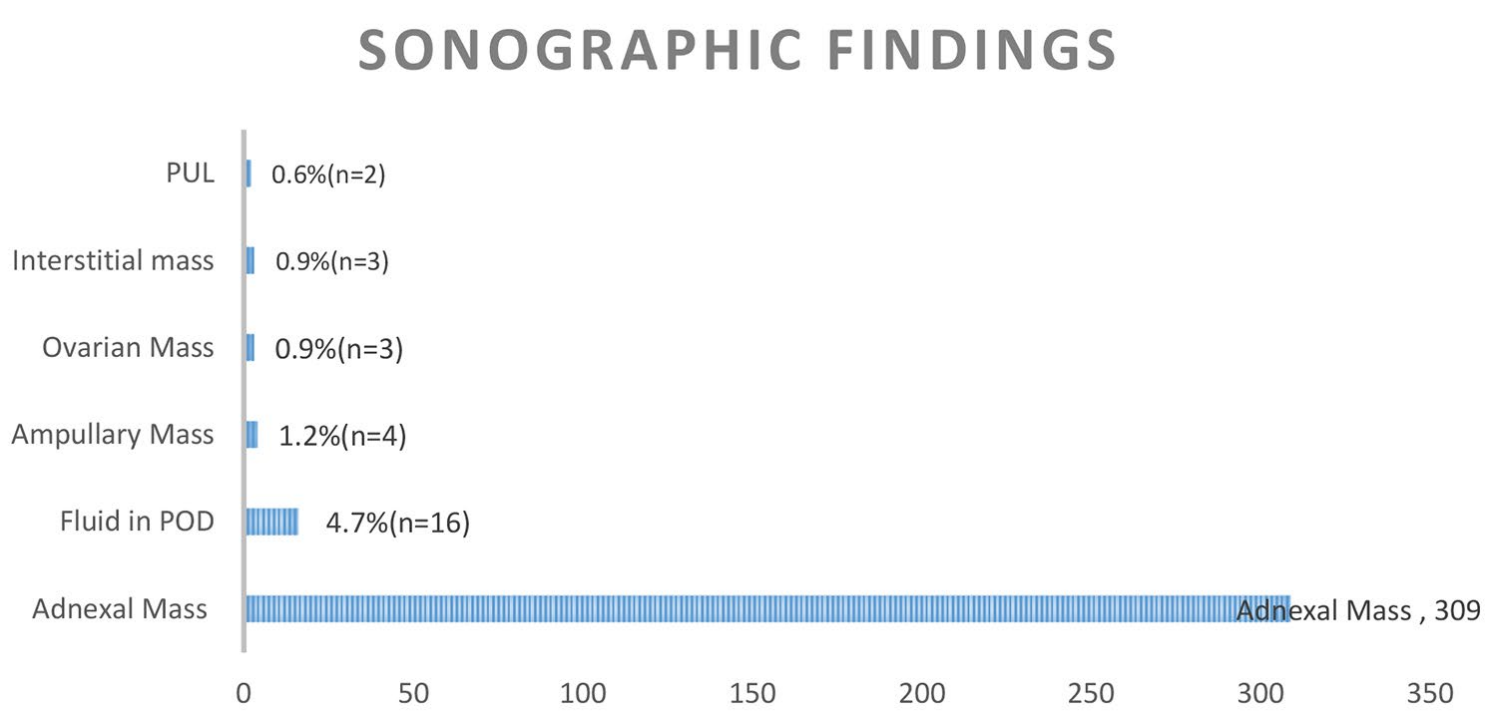
Distribution of sonographic findings in patients with surgically managed EP

During the study period (2011–2020), we recorded 347 cases of surgically managed ectopic pregnancies. A total of 337 patient had sonographic findings which represents 97.1% of the total sample. An adnexal mass was the commonest sonographic finding on ultrasound with 91.7% (*n* = 309) of the scans concluding this finding. Significant free fluid in the Pouch of Douglas (POD), defined as fluid that was more than 30 cubic centimetres in volume, was the second most common finding in 4.7% of the ultrasound scans (*n* = 16). The other findings included: an ampullary mass in 1.2% (*n* = 4); ovarian and interstitial mass each in 0.9% (*n* = 3) and no mass was identified in 0.6% (*n* = 2) of the patients. The patients with no mass on ultrasound were labelled as pregnancy of unknown location (PUL). These finds are shown in Fig. 1 below.

A total of 336 patients had a confirmed ectopic pregnancy during surgery. A total of 11 patients (3.2%) did not have any discernible EP during surgery. In the patients with sonographic findings (*n* = 337), only 1 patient had a negative laparoscopy with no EP identified. This patient had a corpus luteum cyst on the side of the suspected EP and this could have led to the wrong diagnosis of this adnexal mass. This represents less than 0.3% of patients in this study.

On analysis regarding the sonographic location corresponding with the intraoperative location, a total of 4 patients had a sonographic location of an ampullary EP, 75% [3] of these patients confirmed this location on surgery. A total of 3 patients had a suspicion of an ovarian EP on ultrasound. However, none (0%; *n* = 0) of these patients had an ovarian EP but 2 (67%) had an ampullary EP while 1(33%) had an interstitial EP. The 3 patients who had a sonographic finding of an interstitial EP all had interstitial EP (100%).

**Fig. 2:**
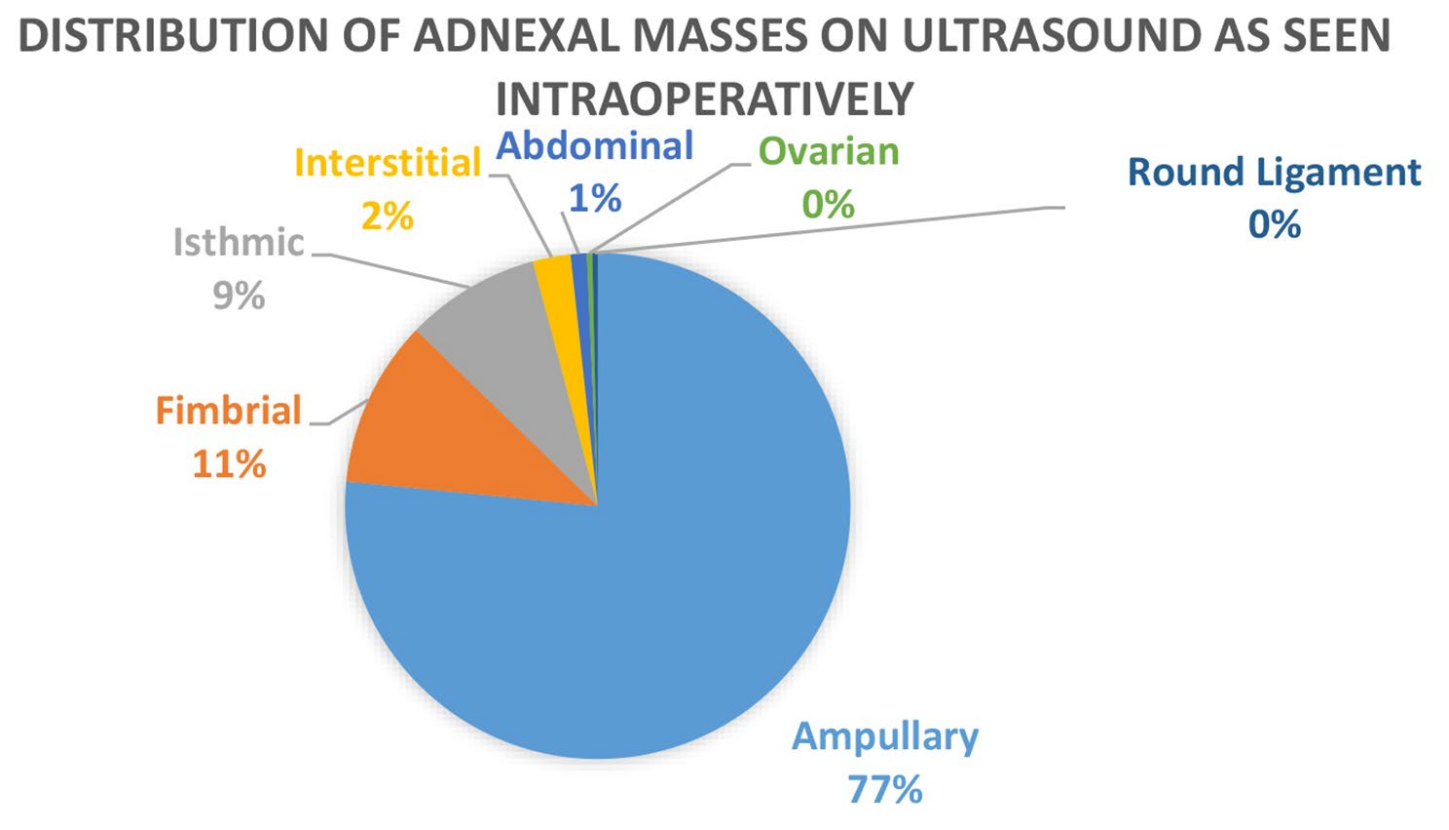
Distribution of Adnexal Masses on Ultrasound with their intraoperative location

A total of 309 Patients had a sonographic finding of an adnexal mass, 290 of these were confirmed surgically at the following locations: 76.6% (*n* = 222) were ampullary in location; 10.7% (*n* = 31) were fimbrial in location; 8.6%(*n* = 25) were isthmic in location; 2.4%(*n* = 7) were interstitial in location; 1%(*n* = 3) were abdominal in location; while 0.3% were located in the ovary(*n* = 1) or round ligament each(*n* = 1). This data is shown in Fig. 2. 15 patients had a finding of free fluid in the POD on ultrasound that on surgery were redistributed as 67%(*n* = 10) were ampullary, 20% (*n* = 3) were fimbrial and 13% (*n* = 2) were interstitial (cornual) in location. The one patient who had a PUL on ultrasound had an ampullary EP.

**Fig. 3:**
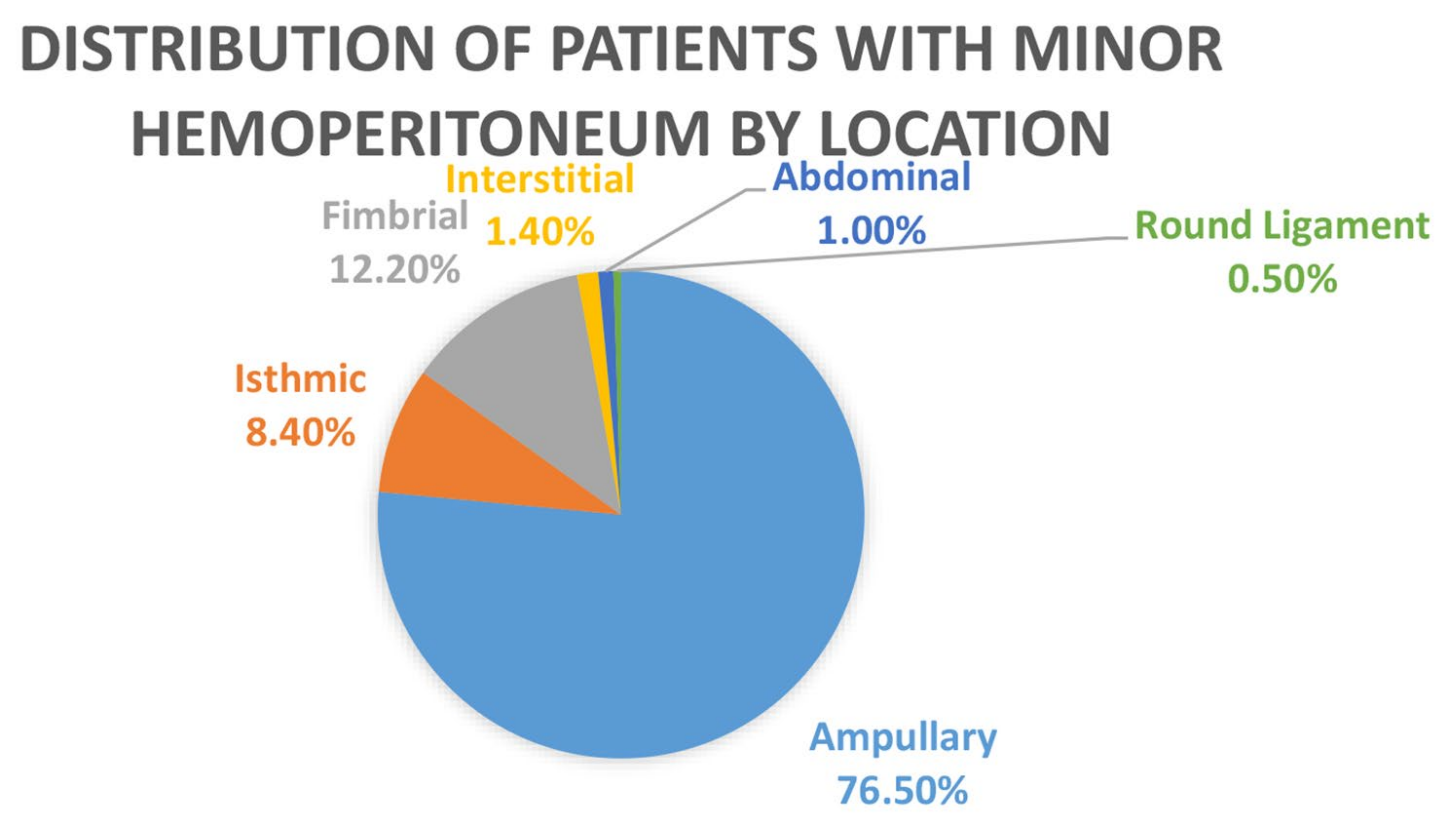
Distribution of patients with minor hemoperitoneum by location

In the analysis of location of EP and whether it could impact on the volume of hemoperitoneum, we subdivided the data into minor hemoperitoneum (< 500mls) or major hemoperitoneum (> 500mls). The patients with minor hemoperitoneum were 213 in number; 76.5%(*n* = 163) of these patients had an ampullary EP. 8.4% (*n* = 18) had an isthmic EP, 12.2% (*n* = 26) had a fimbrial EP, 1.4%(*n* = 3) had a corneal EP, 1% (*n* = 2) had an abdominal EP, 0.5% (*n* = 1) had a round ligament EP and there were no ovarian EP in this group. This information is displayed in Fig. 3.

**Fig. 4:**
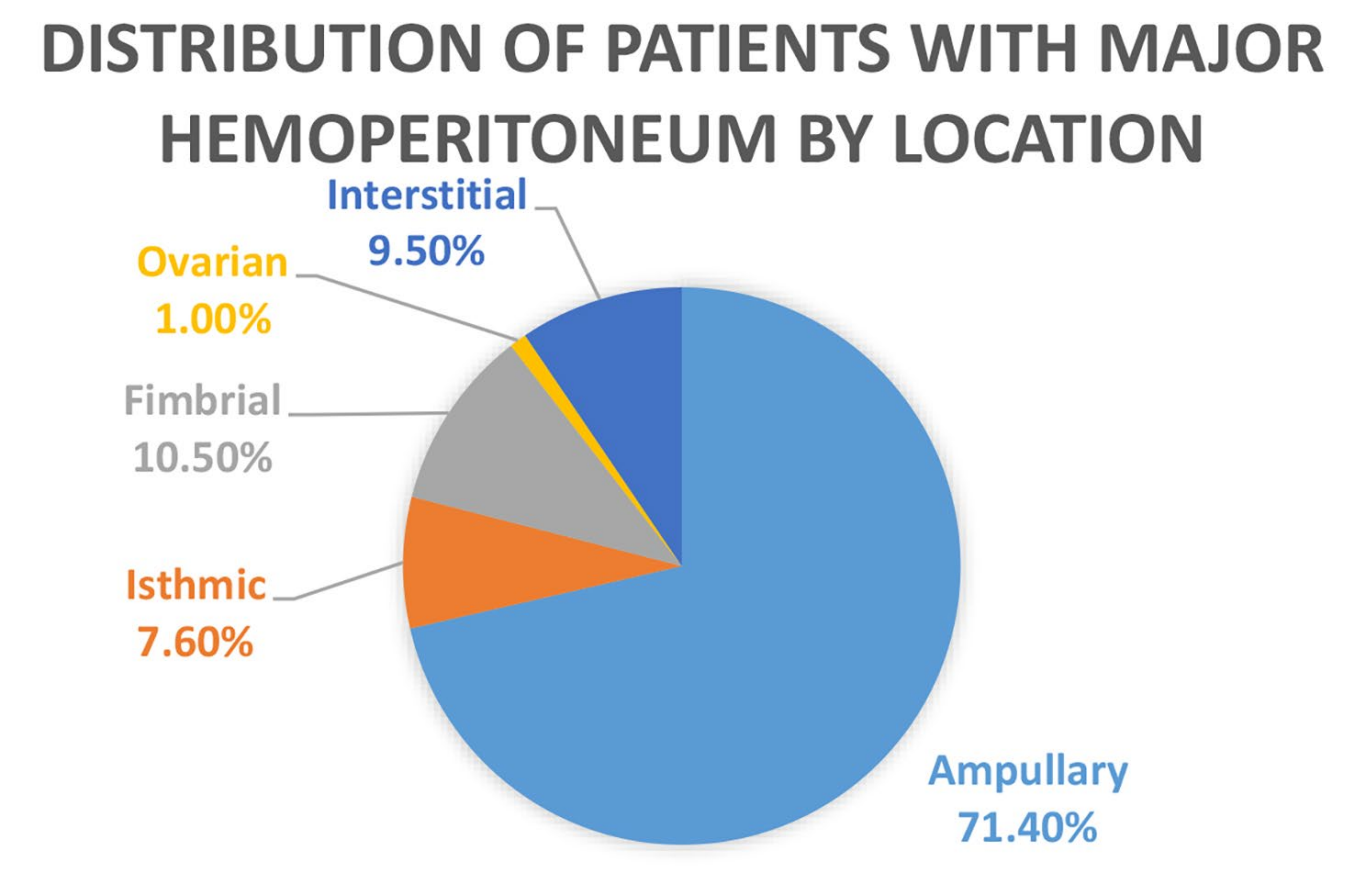
Distribution of patients with major hemoperitoneum by location

The patients with major hemoperitoneum were 105 in number; 71.4% (*n* = 75) of these patients had an ampullary EP. 7.6% (*n* = 8) had an isthmic EP, 10.5% (*n* = 11) had a fimbrial EP, 1%(*n* = 1) had an ovarian EP and 9.5% (*n* = 10) had a interstitial EP and there were no abdominal or round ligament EP in this group. The major significant difference was that corneal EP had a significantly larger proportion of patients in the major hemoperitoneum group compared to the minor hemoperitoneum group (9.5% versus 1.4%). This data is shown in Fig. 4.

Presence of a mass on ultrasound almost invariably corresponded with the presence of an EP at surgery. At total of 139 patient had a mass visualized on ultrasound examination and 97.8% (*n* = 136) of these patients had a confirmed EP at surgery. All patients (*n* = 63; 100%) with a mass size above 38 mm had an EP at surgery.

We further analysed whether the diameter of the EP mass seen on scan could predict the volume of hemoperitoneum and there was no significant correlation (*p* = 0.995, Confidence Interval − 3.0 to 5.584). This meant that increasing size of mass on ultrasound did not predict an increased volume of hemoperitoneum.

## Discussion

EP are an important cause of pregnancy related morbidity and mortality [8]. Their early diagnosis is key with ultrasound examination being central to the investigations required for this to be achieved [5]. We assessed the correlation of sonographic with intraoperative findings among patients with laparoscopically managed EP at a tertiary referral centre.

An adnexal mass was the commonest finding in the present study with 97.1% of the ultrasounds done having this finding. This is in keeping with previous studies that cite this as the major finding in most EP [9]. An adnexal mass with a positive pregnancy test without an intrauterine pregnancy is usually diagnostic of an EP [9]. This is usually the earliest sign even prior to development of a hemoperitoneum [10]. An adnexal mass is seen in around 89–100% of endovaginal ultrasounds done for EP [4]. An adnexal mass can be attributed to the actual pregnancy or an associated hematoma from haemorrhage [9]. In EP that occur in other sites, different ultrasonographic identifiers have been described in literature [11]. Interstitial EP usually have an eccentric location proximal to the uterine cornua with an echogenic line(interstitial line) that may extend from the endometrium to the interstitial mass [12]. Ovarian EP are difficult to identify unless a gestational sac with an embryo surrounded by ovarian tissue is identified [13]. Ovarian EP have also being described using the spiegelberg criteria. These include: a normal uninvolved ipsilateral fallopian tube; the gestational sac being in the normal position of the ovary & the ovary and the gestational sac are connected to the uterus via the ovarian ligament [13].A ceserean scar pregnancy will be sonographically identified by seeing a pregnancy that’s implanted in a prior ceserean scar location usually in the low anterior position closer to the cervix than the uterine fundus [14]. Abdominal pregnancies are usually identified by either a gestational sac or a fetus or placental tissue that’s outside the uterus with no surrounding myometrium between it and other pelvic organs like the urinary bladder [7]. Endovaginal ultrasound is better than transabdominal ultrasound in detecting an EP [15].

337 patients in the present study had a sonographic finding expected with an EP. Only 1 patient had a negative laparoscopy representing a detection rate of 99.7%. this high detection rate is in keeping with previous studies with the ultrasound performing better than most imaging modalities [4, 5, 9]. The use of endovaginal sonography almost virtually eliminates the possibility of an EP being missed [15].

In correlating the sonographic location to the intraoperative one, most patients (91.6%) just had a finding of an adnexal mass with no specific location of the EP. This is an expected trend since ultrasound rarely identifies the specific location of an EP with the finding of an adnexal mass being the most common [9]. In terms of specific location, 75% of patients an ampullary location on ultrasound were confirmed intraoperatively. This is in keeping with studies on the sensitivity of ultrasound localizing ampullary EP pregnancies [11, 16]. This is based on the facts that since this is the most common location of EP then a large proportion of ultrasounds may get this location right by chance. However, none of the patients with an ovarian EP were confirmed at that location intraoperatively. This is a usual phenomenon since most of the ovarian EP rarely appear as EP on ultrasound [17]. They are more often identified as ovarian cysts since majority rarely develop a sonographically discernible gestation sac [17]. All patients who had interstitial EP on scan were confirmed intraoperatively. This high pick up rate is in keeping with previous studies on pickup rate of interstitial EP [10, 12]. This is attributed to the fact that Interstitial EP have specific findings on ultrasound among which the interstitial line finding may be the most sensitive (80%) and specific (98%) of these findings [12].

The distribution of the location of EP in the present study was as follows: ampullary 77%; fimbrial 11%; isthmic 9%, interstitial 2% and abdominal 1%. Ovarian or round ligament EP were the rarest representing only 0.3% each. This trend is in keeping with the natural distribution of EP that expected [18]. This trend is anticipated due to the physiology of fertilization and hence decreasing incidence from that site proximally or distally [9] However, the present study had a lower rate (0.3%) of ovarian EP than expected (3.2%). This may be attributed to the fact that these are random EP in occurrence that may or may not happen as common in a given population [11].

We further analysed whether the volume of hemoperitoneum(HP) had any particular trend with regards to the location of EP.in the regards we classified them under major or minor haemorrhage with the cut off between the two being 500 millilitres(mls) of HP. The most significant disparity occurred in the interstitial EP which only accounted for 1.4% of the patients in the minor HP group compared with 9.5% in the major HP group. This is in keeping with studies of interstitial EP due to the fact that most of them are usually ruptured at diagnosis and hence may have a higher volume of HP expected [19]. In patients with equivocal findings of an interstitial EP, identification of major HP may be a useful adjunct to confirming its location.

### Study limitations

This was a retrospective chart audit; a prospective study would have yielded a better data set. However, due to the nature of the standardized practice at our university hospital, the date set was consistently complete.

## Conclusion

In conclusion, ultrasonography still represents the best imaging modality for EP. The most common finding is usually an adnexal mass with no specific location. Most (99.7%) of the patients with this sonographic finding usually have a confirmed EP. Interstitial EP are the most well localized with ultrasound followed by ampullary EP. Furthermore, the presence of major (> 500mls) hemoperitoneum may act as an adjunct for diagnosis of an interstitial EP.

## Data Availability

The materials used during the current study are available from the corresponding author on reasonable request.

## Abbreviations

AKUH: Aga Khan University Hospital
EP: Ectopic Pregnancies
HCG: Human Chorionic Gonadotrophin
HP: Hemoperitoneum
MLS: Millilitres
SPSS: Statistical Package for Social Sciences
